# Obesity aggravates the role of C‐reactive protein on knee pain: A cross‐sectional analysis with NHANES data

**DOI:** 10.1002/iid3.1371

**Published:** 2024-09-02

**Authors:** Ling Luo, Mingzi Li, Wenlong Huang, Siying Zhang, Jianbo Sun, Bingsong Zhang, Wei Hu, Haibing Yu

**Affiliations:** ^1^ Department of Epidemiology and Medical Statistics, School of Public Health Guangdong Medical University Dongguan Guangdong China; ^2^ The First Dongguan Affiliated Hospital Guangdong Medical University Dongguan Guangdong China; ^3^ Dongguan Key Laboratory of Chronic Noncommunicable Disease Prevention Guangdong Medical University Dongguan Guangdong China; ^4^ Institute of Scientific and Technological Information Nanjing University of Aeronautics and Astronautics Nanjing Jiangsu China; ^5^ Department of Epidemiology, School of Public Health Sun Yat‐Sen University Guangzhou Guangdong China

**Keywords:** C‐reactive protein, knee pain, National Health and Nutrition Examination Survey, obesity

## Abstract

**Objective:**

To examine the relationship between C‐reactive protein (CRP) and knee pain, and further explore whether this association is mediated by obesity.

**Methods:**

The population was derived from 1999 to 2004 National Health and Nutrition Examination Survey. Logistic regression was used to analyze the relationship between CRP and knee pain in three different models, and the linear trend was analyzed. A restricted cubic spline model to assess the nonlinear dose−response relationship between CRP and knee pain. Mediation analyses were used to assess the potential mediating role of obesity. Subgroup analyses and sensitivity analyses were performed to ensure robustness.

**Results:**

Compared with adults with lower CRP (first quartile), those with higher CRP had higher risks of knee pain (odds ratio 1.39, 95% confidence interval 1.12−1.72 in third quartile; 1.56, 1.25−1.95 in fourth quartile) after adjusting for covariates (except body mass index [BMI]), and the proportion mediated by BMI was 76.10% (*p* < .001). BMI and CRP were linear dose−response correlated with knee pain. The odds ratio for those with obesity compared with normal to knee pain was 2.27 (1.42−3.65) in the first quartile of CRP, 1.99 (1.38−2.86) in the second, 2.15 (1.38−3.33) in the third, and 2.92 (1.72−4.97) in the fourth.

**Conclusion:**

Obesity mediated the systemic inflammation results in knee pain in US adults. Moreover, higher BMI was associated with higher knee pain risk in different degree CRP subgroups, supporting an important role of weight loss in reducing knee pain caused by systemic inflammation.

## Graphical abstract

(1) Knee pain caused by knee osteoarthritis has become one of the most common clinical complaints in adults and is a serious public health problem that needs to be solved urgently. (2) Inflammation and weight are both modifiable risk factors for knee pain. (3) Obesity‐mediated systemic inflammation results in knee pain in US adults, and weight loss plays an important role in reducing knee pain caused by systemic inflammation.

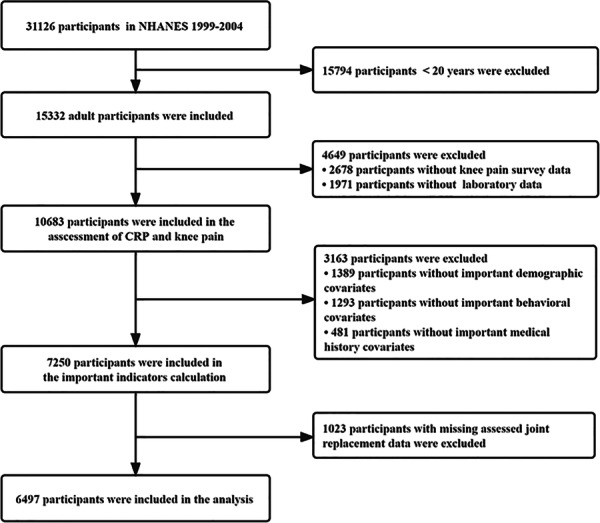

## 1 INTRODUCTION

Chronic pain is an unpleasant sensory and emotional experience associated with, or potential tissue damage, or described in terms of such damage, containing joint pain, with the knee being a frequently affected site.^1^, ^2^ Knee pain is one of the most common clinical complaints in adults, with about half of middle‐aged and elderly people over 50 years old experiencing it.^3^ It is a serious public health problem that needs to be solved urgently, as one in six individuals with knee pain seeks medical attention annually, and a third of them become disabled, causing an enormous burden to individuals and society.^4^

At present, many studies have been conducted to explore the risk factors of knee joint pain, such as race, education level, and marital status.^5^, ^6^, ^7^, ^8^ However, these indicators are often nonmodifiable or difficult to change. Therefore, identifying modifiable risk factors of knee pain is of greater practical value to provide early prevention and intervention opportunities. It is worth noting that among many causes, knee osteoarthritis is the leading cause of knee pain.^9^ Its pathogenesis may be related to the inflammatory response mediated by the synovial membrane and synovial cells present in the joint.^10^ A prospective cohort study showed that systemic inflammation was a predictor of worsening knee pain.^11^ C‐reactive protein (CRP) is a highly sensitive indicator of inflammation and can be a valuable marker for reflecting systemic inflammation.^12^ Furthermore, CRP was found to be significantly associated with different degrees and types of pain in patients with knee pain.^11^, ^13^

Poor lifestyle (drinking, smoking, and lower physical activity) and obesity are strongly associated with systemic inflammation, especially obesity.^6^, ^7^, ^14^, ^15^, ^16^ Higher CRP levels are associated with higher body mass index (BMI).^17^, ^18^ Currently, obesity is recognized as a low‐grade inflammatory disease. At the same time, inflammation is also increasingly recognized as a key factor in the development of obesity.^19^ Several studies have found that higher BMI also was associated with knee pain severity.^14^, ^15^ Therefore, we speculate that the association of CRP with knee pain is mediated through obesity. Reducing knee pain caused by systemic inflammation through weight loss is an effective strategy for preventing knee pain.

To further verify our conjecture, we used data from the National Health and Nutrition Examination Survey (NHANES) to evaluate the association of CRP with knee pain in the US adult population and explore whether this association is mediated by obesity.

## 2 METHODS

### 2.1 Study population

**Figure 1:**
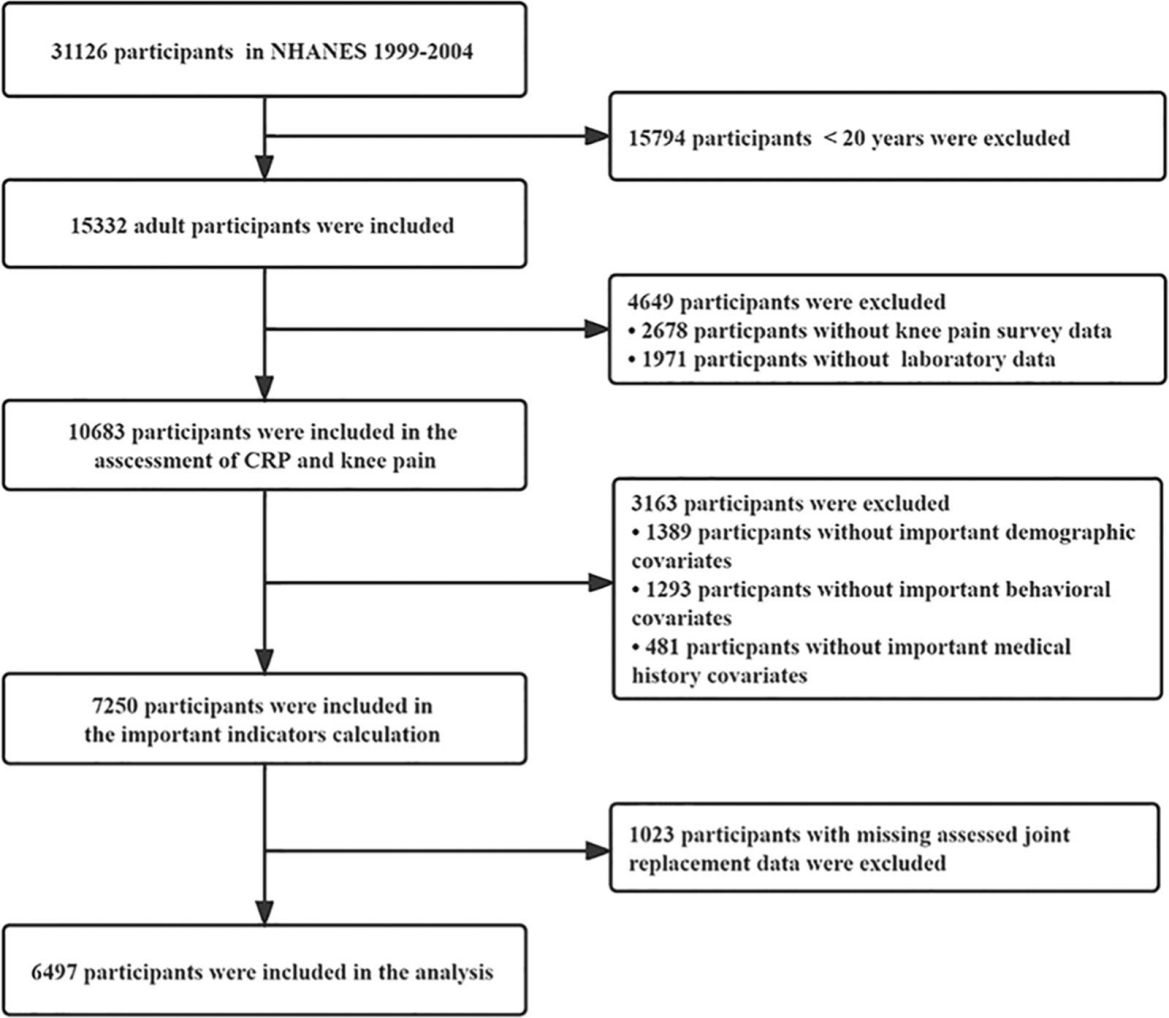
Flowchart of the study participants selection. The NHANES 1999−2004 included a total of 31,126 participants. First, 15,794 participants younger than 20 years were excluded. Second, 2678 and 1971 participants were excluded due to incompleteness data of knee pain and CRP, respectively. Third, 1389 participants without important demographic information (sex, race, educational level, marital status, poverty income ratio, and body mass index), 1293 participants without behavioral information (diet, physical activity, smoking, and alcohol consumption), or 481 participants without medical history data (cardiovascular diseases, diabetes mellitus, hypertension, and stroke) were excluded. Fourth, 1023 participants with missing assessed joint replacements were excluded. Finally, 6497 participants were included in this analysis. CRP, C‐reactive protein; NHANES, National Health and Nutrition Examination Survey.

The population was derived from 1999 to 2004 NHANES, a nationally representative cross‐sectional survey designed and conducted by the National Center for Health Statistics (NCHS).^20^ The NCHS Research Ethics Review Board authorized the survey, verifying that all participants provided informed consent. Detailed statistics are available at https://www.cdc.gov/nchs/nhanes/. We performed an analysis of 31,126 participants in the 1999−2004 NHANES, obtained by using a stratified multistage probability sampling design to obtain a representative sample. Among the 31,126 participants, we excluded (1) those under 20 years old (*n* = 15,794), (2) those with missing survey data about knee pain (*n* = 2678) and laboratory data about CRP (*n* = 1971), (3) those without demographic, behavioral or related disease history information data (*n* = 3163), (4) those with missing assessed joint replacement (*n* = 1023). Overall, 6497 participants were included in the analysis (Figure 1). This study followed STROBE reporting guidelines (Supporting Information Material‐STROBE Checklist).

### 2.2 Assessment of knee pain

Pain status was found in self‐reported personal interview data in Miscellaneous Pain Questionnaire (MPQ) during the NHANES interview, participants are asked a series of questions to assess their joint pain symptoms and pain regions in body. Knee pain was assessed according to the MPQ. Participants were classified as having knee pain if they answered “yes” to the question: “During the past 12 months, had pain, aching, stiffness, or swelling in or around a joint?” and reported right or left knee affected.^21^

### 2.3 Exposure and mediating factors

In our study, the main exposure factor was CRP level, which was quantified by latex nephelometry.^22^ Since the distribution of CRP levels was skewed, natural log‐transformation for CRP (ln‐CRP) was used to normalize the data for the statistical analysis. Continuous and categorical models were used. When ln‐CRP was used as a continuous variable, ln‐CRP after standardization (per 1 standard deviation [SD]) entered the regression model. In the categorical model, the ln‐CRP index was divided into quartiles, with the lowest quartile as the reference group. The ln‐CRP index quartile intervals were [−4.61 to −2.53], [−2.53 to −1.56], [−1.56 to −0.78], and [−0.78 to 3.23]. For mediation analysis, ln‐CRP was z‐normalized.

BMI may play a potential role in the association between CRP and knee pain.^23^, ^24^ For regression models, BMI was divided into normal (<25 kg/m^2^), overweight (25−30 kg/m^2^), and obese (≥30 kg/m^2^) groups.^25^ For mediation analysis, BMI was z‐normalized as a continuous variable.

### 2.4 Covariates

Covariates were based on previous research and considered mainly include demographic variables, behavioral variables, disease history variables, and joint replacement status.^6^, ^7^, ^26^, ^27^, ^28^, ^29^ Demographic variables included age, sex (male or female), educational levels (less than high school, completed high school, or more than high school), race (non‐Hispanic White, non‐Hispanic Black, Mexican American, or other races), marital status (never married, married or living with partner, or divorced, widowed or separated), and family poverty income ratio (PIR). The PIR can be used as a proxy for socioeconomic status, and the lower PIR value, the lower the socioeconomic status.^21^ Behavioral variables included Healthy Eating Index (HEI)‐2015, smoking status (never, former, or now), alcohol use (never, former, mild, moderate, heavy), vigorous‐intensity physical activity (VPA) (no or yes), moderate‐intensity physical activity (MPA) (no or yes), walking or cycling (no or yes), and muscle‐strengthening activities (MSA) (no or yes). The HEI is a tool developed by the United States Department of Agriculture and the National Cancer Institute to evaluate the extent to which diets are consistent with the Dietary Guidelines for Americans.^30^ A higher HEI score reflects healthier eating. PA is known to be significantly associated with knee pain, but different types of PA have different effects on knee pain.^16^, ^31^ To effectively control the confounding interference of PA on the results, we refine PA into VPA, MPA, walking or cycling, and MSA. Besides, inflammatory responses are also a key mechanism in many chronic diseases.^32^ When diabetes mellitus (DM), hypertension, and obesity are combined with knee osteoarthritis, patients will experience more severe pain.^27^, ^33^ Hence, disease history variables included cardiovascular diseases (CVD), DM, hypertension, and stroke (all classified as no or yes). The basis for diagnosis and classification of all covariates can be found in Supporting Information S1: Table S1.

### 2.5 Statistical analysis

All analyses incorporated sampling weights, strata, and primary sampling units to provide reliable national estimates. Continuous variables were reported as weighted mean ± standard error (mean ± SE), and categorical variables were reported as weighted proportions (%). Weighted *t*‐tests, one‐way ANOVA test (continuous variable), and Rao‐Scott chi‐square tests (categorical variables) were utilized to evaluate the differences between knee pain and non‐knee pain participants or in different groups of quartile‐transformed ln‐CRP.

Three statistical models were fitted and a weighted logistic regression was used to estimate odds ratio (OR) and 95% confidence interval (CI) of knee pain in relation to quartiles of ln‐CRP. *p*‐values for linear trend were estimated by modeling the categories of quartile‐/tertile‐transformed baseline exposure variables as continuous variables. We used variables with significant differences between groups as covariates in the model, and after excluding the effects of collinearity, we determined the covariates that were finally included in the regression model. The generalized variance inflation factor (GVIF) was used to identify multicollinearity in the models. The values of GVIF greater than 4 were considered to have multicollinearity.^34^, ^35^ Therefore, race (GVIF = 13.94) and alcohol use (GVIF = 40.34) were removed from the model. Model 1 did not adjust any covariates. Model 2 adjusted for age, marital status, education level, VPA, smoking status, DM, CVD, hypertension, stroke, and joint replacement. Model 3 was additionally adjusted for BMI based on Model 2. We further conducted a stratified analysis by quartiles of ln‐CRP to investigate associations of BMI with the risk of knee pain among adults in different quartiles of ln‐CRP subgroups and analyzed the effect of the interaction between CRP and BMI on knee pain. Besides, using a restricted cubic spline model to assess the nonlinear dose−response relationship between CRP and knee pain. The “mediation” package was used for mediation analysis, with CRP as the independent variable, knee pain as the dependent variable, and BMI as the intermediate variable. Linear regression and logistic regression were used in the mediation and outcome models to evaluate BMI's direct and indirect effects on CRP and knee pain, respectively.

Sensitivity analyses were applied to all models included. Considering that some people who have had joint replacement surgery may experience persistent pain and discomfort, which may affect the judgment of knee pain.^36^, ^37^ Participants with joint replacement were excluded from the primary population (*n* = 6349), and the same analysis methods were performed to ensure the robustness of the results. Besides, the interval of ln‐CRP was re‐divided in tertiles, excluding the influence of different division methods of ln‐CRP interval on the results. All statistical analyses were performed by R 4.0.3 (R Foundation for Statistical Computing, Vienna, Austria). Two‐sided *p *< .05 was considered statistically significant.

## 3 RESULTS

### 3.1 Characteristics of participants

A total of 6497 adult participants were enrolled, 29.39% of whom were considered as having knee pain. Table 1 shows the characteristics of the study population. Compared with non‐knee pain, participants with knee pain were more likely to be older and to have higher proportions of non‐Hispanic White, obesity (BMI ≥ 30 kg/m^2^), former alcohol user, CVD, DM, hypertension, stroke, and joint replacement, less likely to be never married, have high educational level, never smoking, have VPA (*p *< .05). Simultaneously, knee pain sufferers had higher levels of ln‐CRP (*p *< .05).

**Table 1:** Weighted baseline characteristics of the study population.^a^

Characteristics	Total (*n* = 6497)	Non‐knee pain (*n* = 4593)	Knee pain (*n* = 1904)	*χ* ^2^/*t*	*p* Value
Age (years)	43.88 ± 0.32	42.37 ± 0.36	47.40 ± 0.45	9.50	<.001
Sex				1.99	.165
Male	3468 (51.60)	2523 (52.32)	2070 (49.93)		
Female	3029 (48.40)	945 (47.68)	959 (50.07)		
Race				11.40	<.001
Non‐Hispanic White	3201 (72.03)	2150 (70.07)	1053 (76.59)		
Non‐Hispanic Black	1233 (10.05)	875 (10.29)	358 (9.48)		
Mexican American	1548 (7.88)	1189 (9.08)	359 (5.07)		
Others	513 (10.05)	379 (10.55)	134 (8.86)		
Educational level				3.49	.037
Less than high school	1934 (17.33)	1369 (16.92)	565 (18.29)		
Completed high school	1531 (25.85)	1060 (25.16)	471 (27.45)		
More than high school	3032 (56.82)	2164 (57.92)	868 (54.26)		
Marital status				18.41	<.001
Never married	1113 (17.81)	879 (19.66)	234 (13.48)		
Married or living with partner	4117 (65.99)	2909 (65.64)	1208 (66.80)		
Divorced, widowed, or separated	1267 (16.20)	805 (14.70)	462 (19.72)		
PIR	3.06 ± 0.06	3.08 ± 0.06	3.00 ± 0.06	1.87	.069
BMI				82.32	<.001
Normal	2114 (35.06)	1661 (39.41)	453 (24.89)		
Overweight	2365 (34.88)	1694 (35.40)	671 (33.67)		
Obese	2018 (30.06)	1238 (25.19)	780 (41.43)		
HEI	49.47 ± 0.36	49.58 ± 0.39	49.21 ± 0.45	0.87	.387
VPA				4.47	.040
No	4430 (62.39)	3075 (61.48)	1355 (64.50)		
Yes	2067 (37.61)	1518 (38.52)	549 (35.50)		
MPA				0.00	.963
No	3395 (45.47)	2416 (45.50)	979 (45.41)		
Yes	3102 (54.53)	2177 (54.50)	925 (54.59)		
Walking or cycling				0.06	.801
No	4956 (76.51)	3496 (76.41)	1460 (76.76)		
Yes	1541 (23.49)	1097 (23.59)	444 (23.24)		
MSA				1.56	.218
No	4793 (70.20)	3365 (69.67)	1428 (71.45)		
Yes	1704 (29.80)	1228 (30.33)	476 (28.55)		
Alcohol use				4.26	.005
Never	914 (12.08)	669 (12.44)	245 (11.25)		
Former	1214 (15.44)	788 (14.08)	426 (18.62)		
Mild	2098 (34.34)	1473 (34.27)	625 (34.50)		
Moderate	920 (16.36)	662 (16.95)	258 (14.97)		
Heavy	1351 (21.78)	1001 (22.26)	350 (20.66)		
Smoking status				9.13	<.001
Never	3357 (51.35)	2464 (53.14)	893 (47.16)		
Former	1640 (23.57)	1071 (22.20)	569 (26.75)		
Now	1500 (25.09)	1058 (24.65)	442 (26.09)		
DM				39.63	<.001
No	5725 (91.86)	4137 (93.56)	1588 (87.88)		
Yes	772 (8.14)	456 (6.44)	316 (12.12)		
CVD				45.97	<.001
No	6043 (95.12)	4360 (96.46)	1683 (92.00)		
Yes	454 (4.88)	233 (3.54)	221 (8.00)		
Hypertension				52.09	<.001
No	4043 (68.59)	3052 (72.29)	991 (59.94)		
Yes	2454 (31.41)	1541 (27.71)	913 (40.06)		
Stroke				16.29	<.001
No	6354 (98.47)	4519 (98.90)	1835 (97.46)		
Yes	143 (1.53)	74 (1.10)	69 (2.54)		
Joint replacement				52.06	<.001
No	6349 (98.02)	4524 (98.74)	1825 (96.35)		
Yes	148 (1.98)	69 (1.26)	79 (3.65)		
Ln‐CRP (mg/dL)	−1.73 ± 0.02	−1.82 ± 0.03	−1.50 ± 0.04	6.37	<.001
Quartiles of ln‐CRP				16.77	<.001
Q1	1682 (29.14)	1288 (31.75)	394 (23.03)		
Q2	1633 (25.13)	1186 (26.01)	447 (23.09)		
Q3	1559 (23.27)	1066 (21.98)	493 (26.28)		
Q4	1623 (22.46)	1053 (20.25)	570 (27.61)		

Abbreviations: BMI, body mass index; CRP, C‐reaction protein; CVD, cardiovascular disease; DM, diabetes mellitus; HEI, healthy eating index; ln, natural‐logarithm; MPA, moderate‐intensity physical activity; MSA, muscle‐strengthening activities; PIR, poverty impact ratio; VPA, vigorous‐intensity physical activity.

Normal: BMI < 25 kg/m^2^; overweight: 25 ≤ BMI < 30 kg/m^2^; obese: BMI ≥ 30 kg/m^2^.

^a^ Rate and mean ± standard error were weighted; weighted *t*‐test was used for continuous variables; and Rao‐Scott chi‐square test was used for categorical variables.

### 3.2 Characteristics of the participants across quartiles of Ln‐CRP

Characteristics of the study population according to the quartiles of ln‐CRP were shown in Supporting Information S1: Table S2. The higher level of ln‐CRP, the higher the proportion of females, non‐Hispanic Black, education level less than high school, divorced or widowed or separated, obesity, physically inactive, former alcohol user, smoking now, CVD, DM, hypertension, and stroke (*p *< .05). Age also showed an increasing trend (*p *< .05). In contrast, PIR and HEI showed a decreasing trend (*p *< .05).

### 3.3 Association between Ln‐CRP and knee pain in the study population

**Figure 2:**
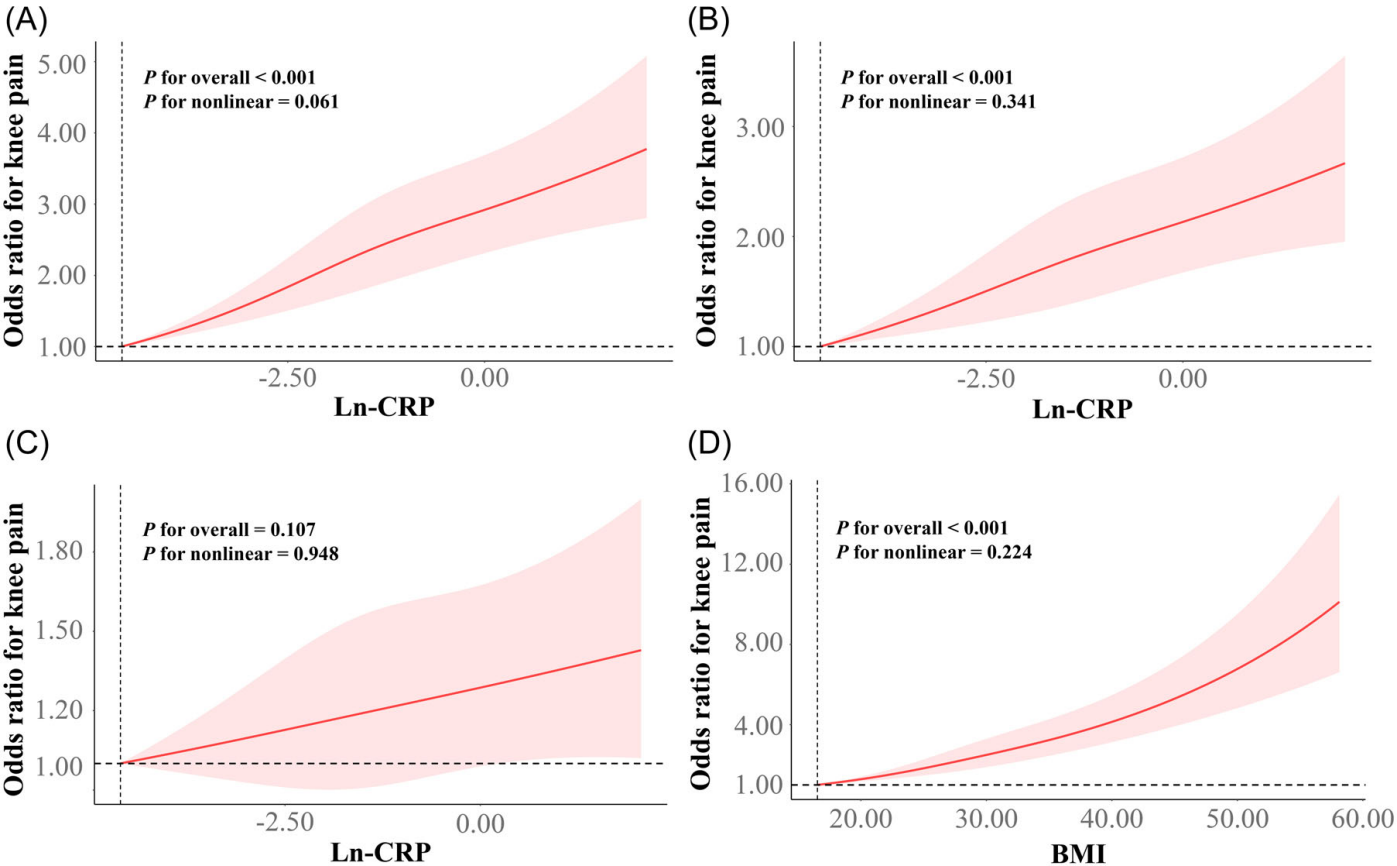
The nonlinear dose−response relationships of ln‐CRP (A−C) and BMI (D) with knee pain in all participants. Results were from restricted cubic spline models; (A) did not adjust any covariates; (B) was adjusted for age, marry, educational level, VPA, smoking status, DM, CVD, hypertension, stroke, and joint replacement; (C) was adjusted for age, marry, educational level, VPA, smoking status, DM, CVD, hypertension, stroke, joint replacement, and BMI; (D) was adjusted for age, marry, educational level, VPA, smoking status, DM, CVD, hypertension, stroke, joint replacement, and ln‐CRP. BMI, body mass index; CRP, C‐reactive protein; CVD, cardiovascular diseases; VPA, vigorous‐intensity physical activity.

As shown in Table 2, compared with first quartile, the third and highest quartiles of ln‐CRP was positively correlated with the risk of knee pain in Model 1 (OR_Q3_ = 1.65, 95% CI: 1.34−2.03; OR_Q4_ = 1.88, 95% CI: 1.53−2.31) and Model 2 (OR_Q3_ = 1.39, 95% CI: 1.12−1.72; OR_Q4_ = 1.56, 95% CI: 1.25−1.95). Moreover, a significant dose−response relationship was found in Model 1 and Model 2 (*p*‐trend < .001). However, all associations disappeared when BMI was added simultaneously in Model 2. Meanwhile, the association between tertiles of ln‐CRP and knee pain had the same characteristics (Supporting Information S1: Table S3). As shown in Figure 2, the spline models confirmed that there was no significant nonlinear relationship between ln‐CRP and the risk of knee pain (*p*_nonlinear_ in all three models were .061, .341, .948, respectively), nor between BMI and knee pain (*p*_nonlinear_ = .224).

**Table 2:** Association between quartiles of ln‐CRP and knee pain in the study population.^a^

Characteristics	Model 1^b^	*p* Value	Model 2^c^	*p* Value	Model 3^d^	*p* Value
	OR (95% CI)		OR (95% CI)		OR (95% CI)	
Quartiles of ln‐CRP						
Q1^e^	Reference		Reference		Reference	
Q2	1.22 (0.99−1.51)	.059	1.09 (0.88−1.35)	.409	0.96 (0.77−1.19)	.685
Q3	1.65 (1.34−2.03)	<.001	1.39 (1.12−1.72)	.004	1.09 (0.85−1.39)	.485
Q4	1.88 (1.53−2.31)	<.001	1.56 (1.25−1.95)	<.001	1.14 (0.90−1.46)	.265
*p*‐trend	<.001		<.001		.198	
BMI						
Normal					Reference	
Overweight					1.36 (1.16−1.60)	<.001
Obese					2.25 (1.81−2.81)	<.001
Per 1 SD^f^	1.30 (1.20−1.41)	<.001	1.21 (1.11−1.32)	<.001	1.07 (0.97−1.18)	.185

Abbreviations: BMI, body mass index; CI, confidence interval; CRP, C‐reaction protein; CVD, cardiovascular diseases; Ln, natural‐logarithm; OR, odds ratio; Q1, the first quartile; Q2, the second quartile; Q3, the third quartile; Q4, the fourth quartile; VPA, vigorous‐intensity physical activity.

Normal: BMI < 25 kg/m^2^; overweight: 25 ≤ BMI < 30 kg/m^2^; obese: BMI ≥ 30 kg/m^2^.

^a^ All estimates were weighted.

^b^ Model 1: Did not adjust any covariates.

^c^ Model 2: Adjusted for age, marry, educational level, VPA, smoking status, DM, CVD, hypertension, stroke, and joint replacement.

^d^ Model 3: Adjusted for age, marry, educational level, VPA, smoking status, DM, CVD, hypertension, stroke, joint replacement, and BMI.

^e^ Q1 was the lowest quartile and was used as the reference in logistic regression analysis.

^f^ Per 1 SD meant OR per one SD increase in logistic regression.

### 3.4 Subgroup analysis

When stratified by quartiles of ln‐CRP, there was no interaction association between BMI and ln‐CRP in Model 1 (*p*_interaction_ = .694) and Model 2 (*p*_interaction_ = .474) (Supporting Information S1: Table S4). And, compared with the normal group, obesity was significantly positively correlated with knee pain both in Model 1 (OR_Q1_ = 2.38, 95% CI: 1.52−3.74; OR_Q2_ = 2.04, 95% CI: 1.42−2.94; OR_Q3_ = 2.13, 95% CI: 1.40−3.22; OR_Q4_ = 2.99, 95% CI: 1.90−4.70) and Model 2 (OR_Q1_ = 2.27, 95% CI: 1.42−3.65; OR_Q2_ = 1.99, 95% CI: 1.38−2.86; OR_Q3_ = 2.15, 95% CI: 1.38−3.33; OR_Q4_ = 2.92, 95% CI: 1.72−4.97). Within each ln‐CRP subgroup, a higher BMI was associated with a higher risk of knee pain (*p*‐trend <.001).

### 3.5 Mediating role of BMI

**Figure 3:**
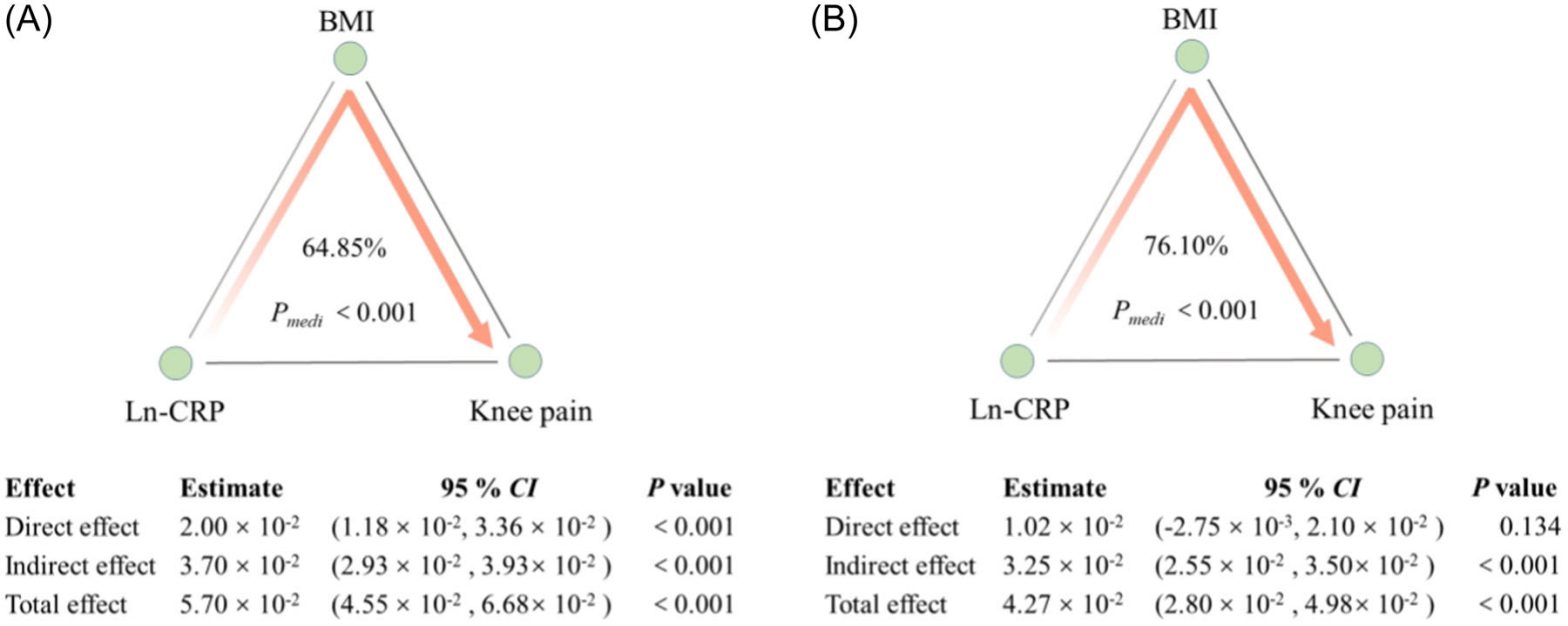
Mediating effect of BMI between ln‐CRP and knee pain in all participants. Both BMI and ln‐CRP are standardized. The 95% CI of these estimates was computed using the bootstrap method (1000 samples). (A) did not adjust any covariates; (B) was adjusted for age, marry, educational level, VPA, smoking status, DM, CVD, hypertension, stroke, and joint replacement. BMI, body mass index; CRP, C‐reactive protein; CVD, cardiovascular diseases; VPA, vigorous‐intensity physical activity.

As shown in Figure 3, increased ln‐CRP was associated with an increased risk of knee pain (OR: 5.70 × 10^−2^, 95% CI: 4.55 × 10^−2^−6.68 × 10^–2^, *p* < .001), and the effect (64.85%) can be explained by a significant indirect effect of BMI (OR: 3.70 × 10^−2^, 95% CI: 2.93 × 10^−2^−3.93 × 10^−2^, *p* < .001) (Figure 3A). After adjusting for covariates, the proportion of indirect effect of BMI increased to 76.10% (OR: 3.25 × 10^−2^, 95% CI: 2.55 × 10^−2^−3.50 × 10^−2^, *p* < .001), and the direct effect of CRP on knee pain became no longer significant (OR: 1.02 × 10^−2^, 95% CI: −2.75 × 10^−3^ to 2.10 × 10^−2^, *p* = .134) (Figure 3B).

### 3.6 Sensitivity analysis

The characteristics of the population, excluding joint replacement were basically similar to the previous population characteristics (Supporting Information S1: Table S5). In the population without joint replacement, compared with first quartile, the third and highest quartiles of ln‐CRP was also positively correlated with the risk of knee pain in Model 1 (OR_Q3_ = 1.63, 95% CI: 1.32−2.00; OR_Q4_ = 1.85, 95% CI: 1.52−2.26) and Model 2 (OR_Q3_ = 1.38, 95% CI: 1.12−1.71; OR_Q4_ = 1.25, 95% CI: 1.25−1.91). Moreover, a significant dose−response relationship was also found in Model 1 and Model 2 (*p‐*trend <.05). Meanwhile, the association between tertiles of ln‐CRP and knee pain had the same characteristics (Supporting Information S1: Table S6). Besides, as shown in Supporting Information S1: Figure S1, there was no significant nonlinear relationship between ln‐CRP and the risk of knee pain (*p*_nonlinear_ in all three models were .066, .338, .913, respectively), nor between BMI and knee pain (*p*_nonlinear_ = .266). Subgroup analysis results also showed that there was no interaction association between BMI and ln‐CRP, and obesity was significantly positively correlated with knee pain among different quartiles of ln‐CRP subgroups (*p* < .05) (Supporting Information S1: Table S7). We also found that after adjusting for covariates, ln‐CRP affected knee pain mainly through the mediating factor BMI (Supporting Information S1: Figure S2).

## 4 DISCUSSION

In this extensive cross‐sectional study, by analyzing the association between CRP and knee pain in adults, and exploring the moderating effect of obesity in this relationship for the first time, we provide an important reference for improving knee pain in real practice by reducing inflammatory responses through weight loss. We observed that elevated CRP levels were positively associated with the risk of knee pain. This is similar to the results of some studies. Among 596 women with hip and/or knee osteoarthritis, ultra‐sensitive CRP level intensity correlated with pain.^38^ Moreover, a prospective cohort study showed that baseline CRP was positively associated with a change in total knee pain (*β* = .33 per mg/L, *p* = .032) and change in CRP was also associated with a change in knee pain at night and when sitting/lying (both *p* < .05) in older adults.^11^ In the cohort hip and cohort knee, CRP was significantly associated with mild nocturnal pain (OR: 1.18, 95% CI: 1.01–1.37), with mild and moderate pain while walking (OR: 1.17, 95% CI: 1.01–1.35 and OR: 1.56, 95% CI: 1.29–1.90, respectively) and with progression of nocturnal pain (OR: 1.25, 95% CI: 1.07–1.46).^13^ However, it can be seen that most of the existing studies were small sample studies, focused on specific groups (the elderly, female, or patients with knee osteoarthritis), or did not specifically focus on knee pain groups. That results in certain limitations in determining the association between CRP and knee pain in the entire population. Therefore, our study has filled the gap to a certain extent by analyzing the relationship between CRP and knee pain in a large, nationally representative sample.

One reason we focused our research on obesity is that obesity is another modifiable risk factor for knee pain. A cross‐sectional study of individuals with knee pain, more than half of included patients were overweight or obese, and 80% had central obesity.^39^ At the same time, a Mendelian randomization study confirmed positive causal associations between BMI on knee pain.^40^ These are similar to our findings, although not exactly the same population we focused on. In our study, more than 75% (weighted) of the participants in the knee pain group were obese or overweight (Table 1). We also found that obese or overweight individuals were significantly positively correlated with knee pain at different quantiles of ln‐CRP (Supporting Information S1: Table S4), and there was a linear dose−response relationship between BMI and knee pain (Figure 2D).

Sylwander et al. believed that overweight/obese individuals have a lower pain threshold and are therefore more susceptible to knee pain.^41^ In a community study, adopting a simple low‐intensity lifestyle reduced the risk of worsening knee pain, especially in women who were overweight or obese.^15^ In addition, studies have found that dietary fiber may reduce knee pain in part by reducing weight and inflammation.^42^ It can be seen that anti‐inflammation and weight loss are effective measures to improve the risk of knee pain. Traditional clinical treatments such as anti‐inflammation and weight loss have been proven effective in relieving knee pain. However, with the advancement and development of science and technology, tissue engineering technology has been widely explored to repair osteochondral loss, using the natural regeneration potential of biomaterials to control cell functions, and ultimately achieve long‐term suppression of pain.^43^ In the future, methods that combine traditional treatments with tissue engineering techniques may have great potential in preventing and treating knee pain.

On the other hand, obesity and inflammation have been extensively linked,^44^ and the logical relationship between weight loss and anti‐inflammation remains unclear in improving knee pain. Multiple meta‐analyses have confirmed that CRP levels were also related with increasing BMI and adiposity.^45^, ^46^ Also, obesity is recognized as a low‐grade inflammatory disease.^17^, ^45^ Gløersen et al. found that higher BMI was associated with the severity of knee pain, but CRP did not mediate this association.^14^ But, in basic research, one of the most plausible explanations for increased prevalence of chronic pain in obese and aging populations could be chronic inflammation.^47^ Besides, evidence from the Arthritis, Diet, and Activity Promotion Trial data showed that inflammatory cytokines mediate the effects of diet and exercise on pain and function in knee osteoarthritis, independent of BMI.^48^ However, the role of BMI in the relationship between CRP and knee pain in people without dietary and exercise intervention has not yet been determined. In our study, subgroup analysis showed that there was no interaction between BMI and CRP (Supporting Information S1: Table S4). We found through mediation analysis that BMI played a partial mediating role in CRP and knee pain, and after adjusting for covariates, BMI became the major mediating role (Figure 3). Therefore, while taking anti‐inflammatory actions to reduce the risk of knee pain, it is also necessary to lose weight, especially in obese people.

The present study is a representative sample of the US population, strictly following well‐designed study protocols, with extensive quality assurance and quality control. Furthermore, a variety of sensitivity analyses demonstrated the robustness for our findings. It must be acknowledged that our study has several limitations. First, causal interpretation is limited due to the design of a cross‐sectional study. Second, knee pain was based on self‐report data, which may underestimate the actual number of knee pain individuals. Finally, since our study is based on the US population, it remains to be determined whether our conclusions apply to other populations.

## 5 CONCLUSION

In conclusion, we leveraged a large, nationally representative data set to reveal for the first time, that obesity exacerbates knee pain in American adults by mediating systemic inflammatory responses. Higher BMI was associated with higher knee pain risk in different degree CRP subgroups, supporting an important role of weight loss in reducing knee pain caused by systemic inflammation. This finding has important clinical implications because it highlights the potential role of weight management in reducing knee pain caused by systemic inflammation and provides a new clinical strategy for early identification and treatment of knee pain through anti‐inflammatory measures.

## AUTHOR CONTRIBUTIONS

**Ling Luo**: Formal analysis; methodology; writing—original draft. **Mingzi Li**: Data curation; methodology; visualization. **Wenlong Huang**: Formal analysis; methodology. **Siying Zhang**: Methodology; visualization. **Jianbo Sun**: Project administration; supervision; validation. **Bingsong Zhang**: Methodology; writing—review and editing. **Wei Hu**: Data curation; methodology; writing—review and editing. **Haibing Yu**: Data curation; funding acquisition; investigation; project administration; writing—review and editing.

## CONFLICT OF INTEREST STATEMENT

The authors declare no conflict of interest.

## ETHICS STATEMENT

The studies involving human participants were reviewed and approved by NCHS IRB/ERB and in accordance with the Helsinki Declaration. The participants provided their written informed consent to participate in this study. Furthermore, all methods were performed following relevant guidelines and regulations. For detailed information, see the following URL: https://www.cdc.gov/nchs/nhanes/irba98.htm.

## Supporting information

Supporting information.

Supporting information.

## Data Availability

The data sets used and/or analyzed in this study are available on the NHANES official website (https://wwwn.cdc.gov/nchs/nhanes/Default.aspx).
