# Supplementary material for: Obesity aggravates the role of C‐reactive protein on knee pain: A cross‐sectional analysis with NHANES data
Source: Immun Inflamm Dis. 2024 Sep 2;12(9):e1371. doi: 10.1002/iid3.1371 (PMC11367918; doi:10.1002/iid3.1371)
Supplement: Supplementary file 1 — Supporting information. [file IID3-12-e1371-s001.docx]

**Table S1** Diagnostic criteria of covariates in NHANES *

| **Characteristics** | **Section** | **English Text** | **Value Description or Value Description for Diagnosis Yes** |
| --- | --- | --- | --- |
| Age | Questionnaire Data | Best age in years of the sample person at time of HH screening. Individuals 85 and over are topcoded at 85 years of age. | Age |
| Sex | Questionnaire Data | Gender of the sample person | Male, female |
| Educational levels | Questionnaire Data | (SP Interview Version) What is the highest grade or level of school {you have/SP has} completed or the highest degree {you have/s/he has} received? | Less than high school [less than 9th grade or 9-11th grade (includes 12th grade with no diploma)]; Completed high school (high school grad/ged or equivalent); More than high school (some college or aa degree or college graduate or above) |
| Race | Questionnaire Data | Recode of reported race and ethnicity information | Non-Hispanic White; non-Hispanic Black; Mexican American; others (other Hispanic and other race - including Multi-Racial) |
| Marital status | Questionnaire Data | Marital Status | Never married; Married or living with partner; Divorced, widowed or separated |
| PIR | Questionnaire Data | Poverty income ratio (PIR) - a ratio of family income to poverty threshold | Low (PIR ≤ 1.0); Median (1.0-3.0); High (> 3.0) |
| HEI | Dietary Interview | Two 24-h dietary recalls were collected using the United States Department of Agriculture’s (USDA’s) Automated Multiple-Pass Method. | 13 dietary components |
| Alcohol use | Questionnaire Data | The next questions are about drinking alcoholic beverages. Included are liquor (such as whiskey or gin), beer, wine, wine coolers, and any other type of alcoholic beverage. In any one year, {have you/has SP} had at least 12 drinks of any type of alcoholic beverage? By a drink, I mean a 12 oz. beer, a 5 oz. glass of wine, or one and half ounces of liquor.  In {your/SP's} entire life, {have you/has he/ has she} had at least 12 drinks of any type of alcoholic beverage?  In the past 12 months, on those days that {you/SP} drank alcoholic beverages, on the average, how many drinks did {you/he/she} have?  In the past 12 months, on how many days did {you/SP} have 5 or more drinks of any alcoholic beverage? PROBE: How many days per week, per month, or per year did {you/SP} have 5 or more drinks in a single day? | Never (had <12 drinks in lifetime); Former (had ≥12 drinks in 1 year and did not drink last year, or did not drink last year but drank ≥ 12 drinks in lifetime); Moderate (≥ 2 drinks per day for female, or ≥ 3 drinks per day for male, or binge drinking ≥ 2 per month); Heavy (≥ 3 drinks per day for female, or ≥ 4 drinks per day for male, or binge drinking on 5 or more days per month); Mild (not meats the above). |
| Smoking status | Questionnaire Data | {Have you/Has SP} smoked at least 100 cigarettes in {your/his/her} entire life?  {Do you/Does SP} now smoke cigarettes | Never (smoked less than 100 cigarettes in life); Former (smoked more than 100 cigarettes in life and smoke not at all now); Now (smoked moth than 100 cigarettes in life and smoke some days or every day) |
| VPA | Questionnaire Data | Over the past 30 days, did you do vigorous activities for at least 10 min that caused heavy sweating or large increases in breathing or heartbeat? | Yes |
| MPA | Questionnaire Data | Over the past 30 days, did you do moderate activities for at least 10 min that cause only light sweating or a slight-to-moderate increase in breathing or heartbeat? | Yes |
| Walking or cycling |  | Over the past 30 days, did you walk or bicycle as part of getting to and from work or school or to do errands? | Yes |
| MSA |  | Over the past 30 days, did you do any PA specifically designed to strengthen your muscles such as lifting weights, push-ups, or sit-ups? | Yes |
| BMI | Examination Data | Body Mass Index (kg/m^2^) | Dividing the measured weight (kg) by the square of the measured height (m) |
| CVD | Questionnaire Data | Has a doctor or other health professional ever told {you/SP} that {you/s/he} . . .had angina, also called angina pectoris? | Angina-Yes |
|  | Questionnaire Data | Has a doctor or other health professional ever told {you/SP} that {you/s/he} . . .had coronary (kor-o-nare-ee) heart disease? | Coronary heart disease-Yes |
|  | Questionnaire Data | Has a doctor or other health professional ever told {you/SP} that {you/s/he} . . .had congestive heart failure? | Congestive heart failure-Yes |
|  | Questionnaire Data | Has a doctor or other health professional ever told {you/SP} that {you/s/he} . . .had a heart attack (also called myocardial infarction (my-o-car-dee-al in-fark-shun))? | Heart attack-Yes |
|  | Questionnaire Data | Has a doctor or other health professional ever told {you/SP} that {you/s/he} . . .had a stroke? | Stroke-Yes |
|  | Questionnaire Data | Has a doctor or other health professional ever told {you/SP} that {you/he/she} had …? | A heart attack / A stroke |
| Hypertension | Questionnaire Data | {Were you/Was SP} told on 2 or more different visits that {you/s/he} had hypertension, also called high blood pressure? | Yes |
|  | Questionnaire Data | {Have you/Has SP} ever been told by a doctor or other health professional that {you/s/he} had hypertension, also called high blood pressure? | Yes |
|  | Questionnaire Data | Use of antihypertensive medication, including angiotensin converting enzyme inhibitors, angiotensin receptor blockers, diuretics, calcium channel blockers, beta blockers, alpha blockers, centrally acting agents, direct vasodilators, aldosterone receptor antagonists, renin inhibitors, other antihypertensive agents. | Use of antihypertensive medication |
|  | Examination Data | Average SBP, average DBP (at least 3 times) | Average SBP ≥ 140 mmHg, average DBP ≥ 90 mmHg |
| DM | Questionnaire Data | The next questions are about specific medical conditions. {Other than during pregnancy, {have you/has SP}/ {Have you/Has SP}} ever been told by a doctor or health professional that {you have/{he/she/SP} has} diabetes or sugar diabetes? | Yes |
|  | Laboratory Data | Glycohemoglobin | > 6.5% |
|  | Laboratory Data | Fasting glucose | ≥ 7.0 mmol/L |
|  | Laboratory Data | random blood glucose | ≥ 11.1 mmol/L |
|  | Laboratory Data | Two Hour Glucose (OGTT) | ≥ 11.1 mmol/L |
|  | Questionnaire Data | Use of diabetes medication or insulin, including biguanides (metformin), sulfonylureas, insulin, thiazolidinediones, dipeptidyl peptidase 4 inhibitors, glucose like peptide-1 receptor agonists, sodium-glucose co-transporter-2 inhibitors, alpha-glucosidase inhibitors, meglitinides, amylin analogs, other antidiabetic agents. | Use of diabetes medication or insulin |
| Stroke | Questionnaire Data | Has a doctor or other health professional ever told {you/SP} that {you/s/he} . . .had a stroke? | Yes |
|  | Questionnaire Data | Has a doctor or other health professional ever told {you/SP} that {you/he/she} had …? | A stroke |
| Joint replacement | Questionnaire Data | Q12. A hip, bone, or joint replacement? | Yes |

Abbreviations: NHANES, National Health and Nutrition Examination Survey; PIR, poverty impact ratio; BMI, body mass index; HEI, healthy eating index; VPA, vigorous-intensity physical activity; MPA, moderate-intensity physical activity; MSA, muscle-strengthening activities; DM, diabetes mellitus; CVD, cardiovascular disease.

**Table S2** Characteristics of the study population according to the quartiles of ln-CRP *

| Characteristics | Quartiles of ln-CRP (mg/dL) | | | | *χ^2^*/*F* | *P* value |
| --- | --- | --- | --- | --- | --- | --- |
|  | Q1  [-4.61, -2.53] | Q2  (-2.53, -1.56] | Q3  (-1.56, -0.78] | Q4  (-0.78, 3.23] |  |  |
| Age (years) | 39.49±0.41 | 44.56±0.56 | 46.15±0.56 | 46.46±0.41 | 12.08 | < 0.001 |
| Sex |  |  |  |  | 58.98 | < 0.001 |
| Male | 1041 (58.80) | 997 (58.65) | 808 (50.47) | 622 (35.55) |  |  |
| Female | 641 (41.20) | 636 (41.35) | 751 (49.53) | 1001 (64.45) |  |  |
| Race |  |  |  |  | 3.22 | 0.004 |
| Non-Hispanic White | 882 (73.87) | 822 (73.36) | 794 (72.90) | 705 (67.24) |  |  |
| Non-Hispanic Black | 295 (8.72) | 282 (9.28) | 273 (9.45) | 383 (13.24) |  |  |
| Mexican American | 361 (7.49) | 396 (8.00) | 379 (7.63) | 412 (8.520 |  |  |
| Others | 144 (9.92) | 133 (9.37) | 113 (10.02) | 123 (11.01) |  |  |
| Educational level |  |  |  |  | 7.23 | < 0.001 |
| Less than high school | 422 (14.40) | 481 (16.32) | 479 (17.85) | 552 (21.72) |  |  |
| Completed high school | 367 (23.86) | 377 (25.10) | 395 (28.10) | 392 (26.94) |  |  |
| More than high school | 893 (61.75) | 755 (58.58) | 685 (54.06) | 679 (51.34) |  |  |
| Marital status |  |  |  |  | 11.93 | < 0.001 |
| Never married | 432 (24.88) | 233 (15.51) | 223 (14.50) | 225 (14.62) |  |  |
| Married or living with partner | 1029 (62.57) | 1086 (68.30) | 1018 (69.09) | 984 (64.63) |  |  |
| Divorced, widowed or separated | 221 (12.55) | 314 (16.19) | 318 (16.41) | 414 (20.75) |  |  |
| PIR | 3.19±0.08 | 3.12±0.07 | 2.98±0.06 | 2.89±0.07 | 3.28 | 0.002 |
| BMI |  |  |  |  | 129.26 | < 0.001 |
| Normal | 948 (58.35) | 535 (34.71) | 358 (22.80) | 273 (17.94) |  |  |
| Overweight | 569 (31.63) | 725 (43.40) | 588 (36.02) | 483 (28.38) |  |  |
| Obese | 165 (10.01) | 373 (21.89) | 613 (41.18) | 867 (53.68) |  |  |
| HEI | 50.40±0.52 | 50.02±0.45 | 49.31±0.49 | 47.82±0.36 | 3.15 | 0.003 |
| VPA |  |  |  |  | 25.93 | < 0.001 |
| No | 967 (52.18) | 1107 (62.50) | 1110 (65.34) | 1246 (72.45) |  |  |
| Yes | 715 (47.82) | 526 (37.50) | 449 (34.66) | 377 (27.55) |  |  |
| MPA |  |  |  |  | 14.39 | < 0.001 |
| No | 782 (38.81) | 862 (46.87) | 818 (45.81) | 933 (52.20) |  |  |
| Yes | 900 (61.19) | 771 (53.13) | 741 (54.19) | 690 (47.80) |  |  |
| Walking or cycling |  |  |  |  | 6.06 | < 0.001 |
| No | 1227 (72.85) | 1231 (76.22) | 1196 (77.05) | 1302 (81.04) |  |  |
| Yes | 455 (27.15) | 402 (23.78) | 363 (22.95) | 321 (18.96) |  |  |
| MSA |  |  |  |  | 50.71 | < 0.001 |
| No | 1050 (59.08) | 1201 (69.70) | 1215 (76.04) | 1327 (79.15) |  |  |
| Yes | 632 (40.92) | 432 (30.30) | 344 (23.96) | 296 (20.85) |  |  |
| Alcohol use |  |  |  |  | 5.64 | < 0.001 |
| Never | 230 (12.59) | 189 (10.03) | 214 (11.38) | 281 (14.45) |  |  |
| Former | 208 (10.06) | 318 (15.31) | 325 (18.11) | 363 (19.78) |  |  |
| Mild | 584 (36.31) | 574 (37.04) | 507 (34.90) | 433 (28.19) |  |  |
| Moderate | 259 (16.97) | 220 (16.38) | 211 (15.13) | 230 (16.80) |  |  |
| Heavy | 401 (24.06) | 332 (21.24) | 302 (20.49) | 316 (20.78) |  |  |
| Smoking status |  |  |  |  | 3.67 | 0.005 |
| Never | 948 (56.73) | 819 (49.92) | 758 (47.98) | 832 (49.46) |  |  |
| Former | 349 (20.14) | 449 (24.47) | 435 (26.25) | 407 (24.21) |  |  |
| Now | 385 (23.13) | 365 (25.61) | 366 (25.77) | 384 (26.33) |  |  |
| DM |  |  |  |  | 33.89 | < 0.001 |
| No | 1582 (96.15) | 1470 (94.20) | 1337 (89.33) | 1336 (86.27) |  |  |
| Yes | 100 (3.85) | 163 (5.80) | 222 (10.67) | 287 (13.73) |  |  |
| CVD |  |  |  |  | 11.70 | < 0.001 |
| No | 1615 (97.33) | 1511 (95.03) | 1448 (94.49) | 1469 (93.04) |  |  |
| Yes | 67 (2.67) | 122 (4.97) | 111 (5.51) | 154 (6.96) |  |  |
| Hypertension |  |  |  |  | 74.57 | < 0.001 |
| No | 1283 (80.12) | 1043 (71.08) | 875 (62.14) | 842 (57.52) |  |  |
| Yes | 399 (19.88) | 590 (28.92) | 684 (37.86) | 781 (42.48) |  |  |
| Stroke |  |  |  |  | 5.56 | 0.002 |
| No | 1666 (99.39) | 1597 (98.56) | 1523 (97.95) | 1568 (97.70) |  |  |
| Yes | 16 (0.61) | 36 (1.44) | 36 (2.05) | 55 (2.30) |  |  |
| Joint replacement |  |  |  |  | 1.21 | 0.307 |
| No | 1657 (98.69) | 1595 (97.76) | 1515 (97.60) | 1582 (97.89) |  |  |
| Yes | 25 (1.31) | 38 (2.24) | 44 (2.40) | 41 (2.11) |  |  |
| Knee pain |  |  |  |  | 16.77 | < 0.001 |
| No | 1288 (76.33) | 1186 (72.48) | 1066 (66.17) | 1053 (63.17) |  |  |
| Yes | 394 (23.67) | 447 (27.52) | 493 (33.83) | 570 (36.83) |  |  |

* Rate and mean ± standard error were weighted; Weighted one-way ANOVA test was used for continuous variables and Rao-Scott chi-square test was used for categorical variables.

Abbreviations: Ln, natural-logarithm; CRP, C-reaction protein; PIR, poverty impact ratio; BMI, body mass index; HEI, healthy eating index; VPA, vigorous-intensity physical activity; MPA, moderate-intensity physical activity; MSA, muscle-strengthening activities; DM, diabetes mellitus; CVD, cardiovascular disease.

**Table S3** Association between tertiles of ln-CRP and knee pain in the study population*

| Characteristics | Model 1^a^ | *P* value | Model 2^b^ | *P* value | Model 3^c^ | *P* value |
| --- | --- | --- | --- | --- | --- | --- |
|  | *OR* (95% *CI*) |  | *OR* (95% *CI*) |  | *OR* (95% *CI*) |  |
| Tertiles of ln-CRP |  |  |  |  |  |  |
| T1 ^#^ | Reference |  | Reference |  | Reference |  |
| T2 | 1.50 (1.27 - 1.77) | < 0.001 | 1.33 (1.12 - 1.58) | 0.002 | 1.13 (0.94 - 1.36) | 0.177 |
| T3 | 1.77 (1.49 - 2.10) | < 0.001 | 1.51 (1.26 - 1.82) | < 0.001 | 1.14 (0.93 - 1.41) | 0.195 |
| *P*-trend | < 0.001 |  | < 0.001 |  | 0.192 |  |
| BMI |  |  |  |  |  |  |
| Normal |  |  |  |  | Reference |  |
| Overweight |  |  |  |  | 1.35 (1.15 - 1.58) | < 0.001 |
| Obese |  |  |  |  | 2.25 (1.81 - 2.79) | < 0.001 |

* All estimates were weighted.

Abbreviations: Ln, natural-logarithm; CRP, C-reaction protein; *OR*, odds ratio; *CI*, confidence interval.

Normal: BMI < 25 kg/m^2^; Overweight: 25 ≤ BMI < 30 kg/m^2^; Obese: BMI ≥ 30 kg/m^2^.

^a^ Model 1: did not adjust any covariates;

^b^ Model 2: adjusted for age, marry, educational level, VPA, smoking status, DM, CVD, hypertension, stroke, and joint replacement.

^c^ Model 3: adjusted for age, marry, educational level, VPA, smoking status, DM, CVD, hypertension, stroke, joint replacement, and BMI.

**Table S4** Association of BMI with risk of knee pain according by ln-CRP in the study population.

| **BMI** | Model 1^a^ | *P* value | Model 2^b^ | *P* value |
| --- | --- | --- | --- | --- |
|  | *OR* (95% *CI*) |  | *OR* (95% *CI*) |  |
| *P* interaction | 0.694 |  | 0.474 |  |
| Q1 of ln-CRP |  |  |  |  |
| Normal^#^ | Reference |  | Reference |  |
| Overweight | 1.55 (1.20 - 1.99) | 0.001 | 1.46 (1.13 - 1.88) | 0.006 |
| Obese | 2.38 (1.52 - 3.74) | < 0.001 | 2.27 (1.42 - 3.65) | 0.001 |
| *P*-trend | < 0.001 |  | < 0.001 |  |
| Q2 of ln-CRP |  |  |  |  |
| Normal ^#^ | Reference |  | Reference |  |
| Overweight | 1.24 (0.95 - 1.61) | 0.108 | 1.16 (0.88 - 1.53) | 0.284 |
| Obese | 2.04 (1.42 - 2.94) | < 0.001 | 1.99 (1.38 - 2.86) | < 0.001 |
| *P*-trend | < 0.001 |  | < 0.001 |  |
| Q3 of ln-CRP |  |  |  |  |
| Normal ^#^ | Reference |  | Reference |  |
| Overweight | 1.29 (0.88 - 1.90) | 0.189 | 1.26 (0.84 - 1.88) | 0.251 |
| Obese | 2.13 (1.40 - 3.22) | < 0.001 | 2.15 (1.38 - 3.33) | < 0.001 |
| *P*-trend | < 0.001 |  | < 0.001 |  |
| Q4 of ln-CRP |  |  |  |  |
| Normal ^#^ | Reference |  | Reference |  |
| Overweight | 1.93 (1.13 - 3.28) | 0.017 | 1.85 (1.03 - 3.32) | 0.040 |
| Obese | 2.99 (1.90 - 4.70) | < 0.001 | 2.92 (1.72 - 4.97) | < 0.001 |
| *P*-trend | < 0.001 |  | < 0.001 |  |

Abbreviations: BMI, body mass index; Ln, natural-logarithm; CRP, C-reaction protein; *OR*, odds ratio; *CI*, confidence interval; Q1, the first quartile; Q2, the second quartile; Q3, the third quartile; Q4, the fourth quartile.

Normal: BMI < 25 kg/m^2^; Overweight: 25 ≤ BMI < 30 kg/m^2^; Obese: BMI ≥ 30 kg/m^2^; Q1 was the lowest quartile of ln-CRP.

^#^ Normal was used as the reference in logistic regression analysis.

^a^ Model 1: did not adjust any covariates;

^b^ Model 2: adjusted for age, marry, educational level, VPA, smoking status, DM, CVD, hypertension, stroke, and joint replacement.

**Table S5** Weighted baseline characteristics of the population without joint replacement*

| Characteristics | Total | Non-knee pain | Knee pain | *χ^2^*/*t* | *P* value |
| --- | --- | --- | --- | --- | --- |
|  | (n=6349) | (n=4524) | (n=1825) |  |  |
| Age (years) | 43.63±0.32 | 42.20±0.35 | 47.05±0.46 | 9.07 | < 0.001 |
| Sex |  |  |  | 2.28 | 0.138 |
| Male | 3390 (51.57) | 2485 (52.34) | 905 (49.73) |  |  |
| Female | 2959 (48.43) | 2039 (47.66) | 920 (50.27) |  |  |
| Race |  |  |  | 10.68 | < 0.001 |
| Non-Hispanic White | 3113 (71.77) | 2110 (69.87) | 1003 (76.32) |  |  |
| Non-Hispanic Black | 1210 (10.11) | 863 (10.31) | 347 (9.61) |  |  |
| Mexican American | 1519 (7.96) | 1173 (9.14) | 346 (5.14) |  |  |
| Others | 507 (10.16) | 378 (10.67) | 129 (8.92) |  |  |
| Educational level |  |  |  | 2.99 | 0.058 |
| Less than high school | 1868 (17.09) | 1330 (16.65) | 538 (18.14) |  |  |
| Completed high school | 1489 (25.66) | 1046 (25.12) | 443 (26.98) |  |  |
| More than high school | 2992 (57.25) | 2148 (58.23) | 844 (54.88) |  |  |
| Marital status |  |  |  | 17.17 | < 0.001 |
| Never married | 1106 (18.05) | 876 (19.87) | 230 (13.67) |  |  |
| Married or living with partner | 4025 (65.84) | 2867 (65.50) | 1158 (66.64) |  |  |
| Divorced, widowed or separated | 1218 (16.12) | 781 (14.62) | 437 (19.69) |  |  |
| PIR | 3.06±0.06 | 3.09±0.06 | 3.00±0.06 | 1.94 | 0.059 |
| BMI |  |  |  | 77.53 | < 0.001 |
| Normal | 2074 (35.13) | 1637 (39.35) | 437 (25.00) |  |  |
| Overweight | 2325 (35.08) | 1676 (35.55) | 649 (33.96) |  |  |
| Obese | 1950 (29.79) | 1211 (25.10) | 739 (41.04) |  |  |
| HEI | 49.47±0.36 | 49.57±0.40 | 49.23±0.44 | 0.80 | 0.430 |
| VPA |  |  |  | 4.39 | 0.042 |
| No | 4312 (62.27) | 3018 (61.43) | 1506 (64.27) |  |  |
| Yes | 2037 (37.73) | 1294 (38.57) | 531 (35.73) |  |  |
| MPA |  |  |  | 0.00 | 0.989 |
| No | 3308 (45.35) | 2368 (45.35) | 940 (45.33) |  |  |
| Yes | 3041 (54.65) | 2156 (54.65) | 885 (54.67) |  |  |
| Walking or cycling |  |  |  | 0.166 | 0.686 |
| No | 4838 (76.48) | 3439 (76.32) | 1399 (76.86) |  |  |
| Yes | 1511 (23.52) | 1085 (23.68) | 426 (23.14) |  |  |
| MSA |  |  |  | 1.76 | 0.192 |
| No | 4677 (70.13) | 3311 (69.58) | 1366 (71.44) |  |  |
| Yes | 1672 (29.87) | 1213 (30.42) | 459 (28.56) |  |  |
| Alcohol use |  |  |  | 3.25 | 0.020 |
| Never | 890 (12.02) | 659 (12.42) | 231 (11.07) |  |  |
| Former | 1158 (15.13) | 767 (13.98) | 391 (17.88) |  |  |
| Mild | 2054 (34.35) | 1450 (34.22) | 604 (34.65) |  |  |
| Moderate | 909 (16.46) | 655 (16.97) | 254 (15.24) |  |  |
| Heavy | 1338 (22.04) | 993 (22.41) | 345 (21.16) |  |  |
| Smoking status |  |  |  | 8.02 | < 0.001 |
| Never | 3290 (51.49) | 2431 (53.26) | 859 (47.26) |  |  |
| Former | 1588 (23.46) | 1050 (22.22) | 538 (26.45) |  |  |
| Now | 1471 (25.04) | 1043 (24.52) | 428 (26.29) |  |  |
| DM |  |  |  | 33.25 | < 0.001 |
| No | 5608 (91.98) | 4080 (93.57) | 1528 (88.16) |  |  |
| Yes | 741 (8.02) | 444 (6.43) | 297 (11.84) |  |  |
| CVD |  |  |  | 45.82 | < 0.001 |
| No | 5925 (95.38) | 4302 (96.64) | 1623 (92.37) |  |  |
| Yes | 424 (4.62) | 222 (3.36) | 202 (7.63) |  |  |
| Hypertension |  |  |  | 50.03 | < 0.001 |
| No | 3984 (68.89) | 3021 (72.38) | 963 (60.54) |  |  |
| Yes | 2365 (31.11) | 1503 (27.62) | 862 (39.46) |  |  |
| Stroke |  |  |  | 13.47 | < 0.001 |
| No | 6217 (98.58) | 4454 (98.98) | 1763 (97.63) |  |  |
| Yes | 132 (1.42) | 70 (1.02) | 62 (2.37) |  |  |
| Ln-CRP (mg/dL) | -1.73±0.02 | -1.83±0.02 | -1.50±0.05 | 6.45 | < 0.001 |
| Tertiles of ln-CRP |  |  |  | 26.51 | < 0.001 |
| T1 | 2240 (39.08) | 1719 (42.41) | 521 (31.10) |  |  |
| T2 | 2046 (31.17) | 1438 (30.23) | 608 (33.42) |  |  |
| T3 | 2063 (29.76) | 1367 (27.37) | 696 (35.48) |  |  |
| Quartiles of ln-CRP |  |  |  | 16.23 | < 0.001 |
| Q1 | 1657 (29.34) | 1271 (31.82) | 386 (23.38) |  |  |
| Q2 | 1595 (25.06) | 1168 (25.98) | 427 (22.87) |  |  |
| Q3 | 1515 (23.17) | 1048 (21.92) | 467 (26.17) |  |  |
| Q4 | 1582 (22.43) | 1037 (20.28) | 545 (27.58) |  |  |

* Rate and mean ± standard error were weighted; Weighted *t*-test was used for continuous variables and Rao-Scott chi-square test was used for categorical variables.

Abbreviations: PIR, poverty impact ratio; BMI, body mass index; HEI, healthy eating index; VPA, vigorous-intensity physical activity; MPA, moderate-intensity physical activity; MSA, muscle-strengthening activities; DM, diabetes mellitus; CVD, cardiovascular disease; ln, natural-logarithm; CRP, C-reaction protein.

**Table S6** Association between ln-CRP and knee pain in the population without joint replacement *

| Characteristics | Model 1^a^ | *P* value | Model 2^b^ | *P* value | Model 3^c^ | *P* value |
| --- | --- | --- | --- | --- | --- | --- |
|  | *OR* (95% *CI*) |  | *OR* (95% *CI*) |  | *OR* (95% *CI*) |  |
| **Tertiles of ln-CRP** |  |  |  |  |  |  |
| T1^#^ | Reference |  | Reference |  | Reference |  |
| T2 | 1.51 (1.28 - 1.78) | < 0.001 | 1.35 (1.14 - 1.60) | 0.001 | 1.15 (0.95 - 1.38) | 0.139 |
| T3 | 1.77 (1.48 - 2.11) | < 0.001 | 1.51 (1.26 - 1.82) | <0.001 | 1.15 (0.93 - 1.42) | 0.197 |
| *P*-trend | < 0.001 |  | < 0.001 |  | 0.195 |  |
| BMI |  |  |  |  |  |  |
| Normal |  |  |  |  | Reference |  |
| Overweight |  |  |  |  | 1.33 (1.14 - 1.56) | < 0.001 |
| Obese |  |  |  |  | 2.25 (1.81 - 2.79) | < 0.001 |
| **Quartiles of ln-CRP** |  |  |  |  |  |  |
| Q1^#^ | Reference |  | Reference |  | Reference |  |
| Q2 | 1.20 (0.97 - 1.48) | 0.096 | 1.07 (0.86 - 1.33) | 0.514 | 0.94 (0.76 - 1.17) | 0.584 |
| Q3 | 1.63 (1.32 - 2.00) | < 0.001 | 1.38 (1.12 - 1.71) | 0.004 | 1.08 (0.85 - 1.37) | 0.494 |
| Q4 | 1.85 (1.52 - 2.26) | < 0.001 | 1.54 (1.25 - 1.91) | <0.001 | 1.13 (0.89 - 1.43) | 0.307 |
| *P*-trend | < 0.001 |  | < 0.001 |  | 0.213 |  |
| BMI |  |  |  |  |  |  |
| Normal |  |  |  |  | Reference |  |
| Overweight |  |  |  |  | 1.36 (1.15 - 1.59) | < 0.001 |
| Obese |  |  |  |  | 2.26 (1.82 - 2.82) | < 0.001 |
| **Per 1 SD ^d^** | 1.30 (1.20 - 1.41) | < 0.001 | 1.21 (1.11 - 1.32) | < 0.001 | 1.07 (0.97 - 1.19) | 0.168 |

* All estimates were weighted.

Abbreviations: Ln, natural-logarithm; CRP, C-reaction protein; OR, odds ratio; CI, confidence interval; T1, the first tertile; T2, the second tertile; T3, the third tertile; Q1, the first quartile; Q2, the second quartile; Q3, the third quartile; Q4, the fourth quartile.

Normal: BMI < 25 kg/m^2^; Overweight: 25 ≤ BMI < 30 kg/m^2^; Obese: BMI ≥ 30 kg/m^2^. T1 or Q1 was the lowest tertile or quartile of ln-CRP, respectively.

^#^ As the reference in logistic regression analysis.

^a^ Model 1: did not adjust any covariates;

^b^ Model 2: adjusted for age, marry, educational level, VPA, smoking status, DM, CVD, hypertension, and stroke.

^c^ Model 3: adjusted for age, marry, educational level, VPA, smoking status, DM, CVD, hypertension, stroke, and BMI.

^d^ Per 1 SD meant OR per one SD increase in logistic regression.

**Table S7** Association of BMI with risk of knee pain according by ln-CRP in the population without joint replacement

| **BMI** | Model 1^a^ | *P* value | Model 2^b^ | *P* value |
| --- | --- | --- | --- | --- |
|  | *OR* (95% *CI*) |  | *OR* (95% *CI*) |  |
| *P* interaction | 0.577 |  | 0.356 |  |
| Q1 of ln-CRP |  |  |  |  |
| Normal ^#^ | Reference |  | Reference |  |
| Overweight | 1.49 (1.15 - 1.94) | 0.004 | 1.39 (1.07 - 1.81) | 0.015 |
| Obese | 2.29 (1.49 - 3.54) | < 0.001 | 2.18 (1.37 - 3.45) | 0.002 |
| *P*-trend | < 0.001 |  | < 0.001 |  |
| Q2 of ln-CRP |  |  |  |  |
| Normal ^#^ | Reference |  | Reference |  |
| Overweight | 1.29 (0.98 - 1.70) | 0.069 | 1.19 (0.90 - 1.57) | 0.222 |
| Obese | 2.09 (1.45 - 3.01) | < 0.001 | 2.01 (1.40 - 2.88) | < 0.001 |
| *P*-trend | < 0.001 |  | < 0.001 |  |
| Q3 of ln-CRP |  |  |  |  |
| Normal ^#^ | Reference |  | Reference |  |
| Overweight | 1.31 (0.89 - 1.93) | 0.169 | 1.29(0.86 - 1.94) | 0.205 |
| Obese | 2.08 (1.38 - 3.15) | < 0.001 | 2.19(1.41 - 3.39) | < 0.001 |
| *P*-trend | < 0.001 |  | < 0.001 |  |
| Q4 of ln-CRP |  |  |  |  |
| Normal ^#^ | Reference |  | Reference |  |
| Overweight | 1.93 (1.13 - 3.30) | 0.0169 | 1.80 (1.01 - 3.22) | 0.046 |
| Obese | 3.03 (1.91 - 4.80) | < 0.001 | 2.95 (1.74 - 5.00) | < 0.001 |
| *P*-trend | < 0.001 |  | < 0.001 |  |

Abbreviations: BMI, body mass index; Ln, natural-logarithm; CRP, C-reaction protein; *OR*, odds ratio; *CI*, confidence interval; Q1, the first quartile; Q2, the second quartile; Q3, the third quartile; Q4, the fourth quartile.

Normal: BMI < 25 kg/m^2^; Overweight: 25 ≤ BMI < 30 kg/m^2^; Obese: BMI ≥ 30 kg/m^2^; Q1 was the lowest quartile of ln-CRP.

^#^ Normal was used as the reference in logistic regression analysis.

^a^ Model 1: did not adjust any covariates;

^b^ Model 2: adjusted for age, marry, educational level, VPA, smoking status, DM, CVD, hypertension, stroke, and joint replacement.


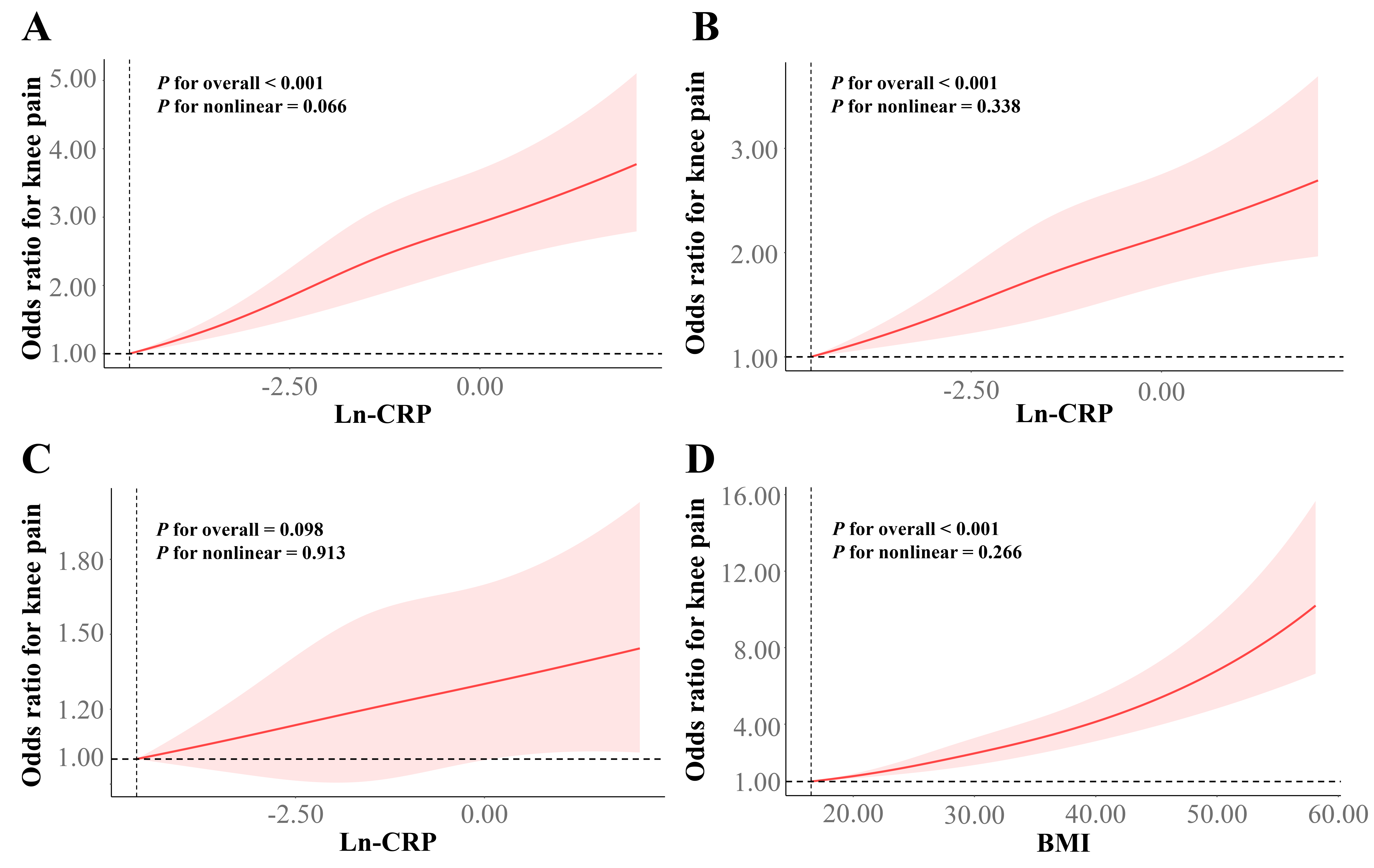


**Figure S1** The nonlinear dose-response relationships of ln-CRP (A–C) and BMI (D) with knee pain in the participants without joint replacement.

**Figure S1 legend** Results were from restricted cubic spline models; A did not adjust any covariates; B was adjusted for age, marry, educational level, VPA, smoking status, DM, CVD, hypertension, stroke, and joint replacement.; C was adjusted for age, marry, educational level, VPA, smoking status, DM, CVD, hypertension, stroke, joint replacement, and BMI. D was adjusted for age, marry, educational level, VPA, smoking status, DM, CVD, hypertension, stroke, joint replacement, and ln-CRP.


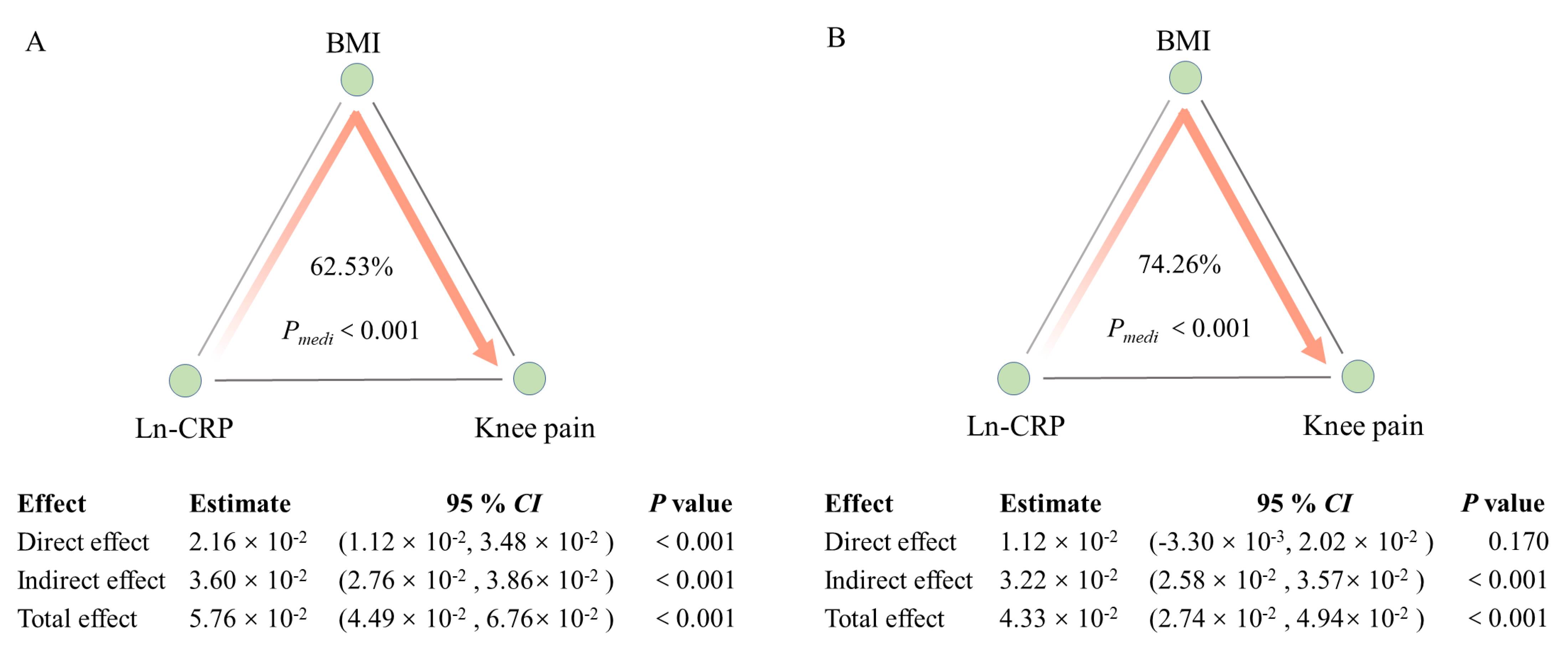


**Figure S2** Mediating effect of BMI between ln-CRP and knee pain in the participants without joint replacement.

**Figure S2 legend** Both BMI and ln-CRP are standardized. The 95% *CI* of these estimates was computed using the bootstrap method (1000 samples). A did not adjust any covariates; B was adjusted for age, marry, educational level, VPA, smoking status, DM, CVD, hypertension, and stroke.
